# Avoidance of reporter assay distortions from fused dual reporters

**DOI:** 10.1261/rna.061051.117

**Published:** 2017-08

**Authors:** Gary Loughran, Michael T. Howard, Andrew E. Firth, John F. Atkins

**Affiliations:** 1 School of Biochemistry and Cell Biology, University College Cork, Cork T12 YT57, Ireland; 2 Department of Human Genetics, University of Utah, Salt Lake City, Utah 84112, USA; 3 Division of Virology, Department of Pathology, University of Cambridge, Cambridge CB2 1QP, United Kingdom

**Keywords:** recoding, frameshifting, readthrough, dual-luciferase, StopGo

## Abstract

Positioning test sequences between fused reporters permits monitoring of both translation levels and framing, before and after the test sequence. Many studies, including those on recoding such as productive ribosomal frameshifting and stop codon readthrough, use distinguishable luciferases or fluorescent proteins as reporters. Occasional distortions, due to test sequence product interference with the individual reporter activities or stabilities, are here shown to be avoidable by the introduction of tandem StopGo sequences (2A) flanking the test sequence. Using this new vector system (pSGDluc), we provide evidence for the use of a 3′ stem–loop stimulator for *ACP2* readthrough, but failed to detect the reported *VEGFA* readthrough.

## INTRODUCTION

Monitoring the translational recoding properties of a test sequence is frequently accomplished by fusing its coding sequence to flanking sequences encoding different reporters, especially where both reporters can be distinguishably assayed in the same tube. Though numerous studies use dual fluorescent reporters for this purpose (Cardno et al. 2009), the advantage of systems with single tube assays was initially shown with dual luciferases (Grentzmann et al. 1998), and these are currently widely used for this purpose. Not all studies check by immunoblotting for possible distortions of the separate reporter activities/stabilities due to a fused test sequence encoded product. We illustrate here vector design features that avoid such distortions by allowing uninterrupted continuous translation of unfused reporters. In theory, this could be accomplished by positioning intein sequences at the 5′ and 3′ ends of the test sequence, since they would result in cleavage of the fusion protein to yield the desired separate proteins. However, as their encoding sequences are not small (100–800 amino acids), we instead use similarly positioned StopGo sequences, which are only 33 amino acids. With StopGo, a conformational change, due to interaction of a specific nascent peptide sequence encoded 5′ of Pro-Gly-Pro with the ribosome peptide exit tunnel, leads to hydrolysis of the ester linkage of peptidyl tRNA^Gly^ such that the product encoded upstream of the underlined Pro codon is released. Translation continues and yields a downstream encoded product whose N terminus is specified by the underlined Pro codon (Ryan and Drew 1994; Donnelly et al. 2001; Doronina et al. 2008; Brown and Ryan 2010). (This phenomenon was initially termed 2A to reflect its discovery in the 2A gene of foot and mouth disease virus [FMDV], but is now variably known as Stop-Carry On [Brown and Ryan 2010], or StopGo [Atkins et al. 2007].) StopGo has been used extensively for both research and biotechnology purposes to achieve highly efficient coexpression of different proteins (Luke et al. 2010; Wang et al. 2013). Using StopGo sequences, the product separation of the upstream encoded reporter occurs as its synthesis is being completed. We illustrate their utility for avoidance of spurious assay values with sequence cassettes whose decoding involves stop codon readthrough.

## RESULTS AND DISCUSSION

### Generating pSGDluc

**FIGURE 1.**
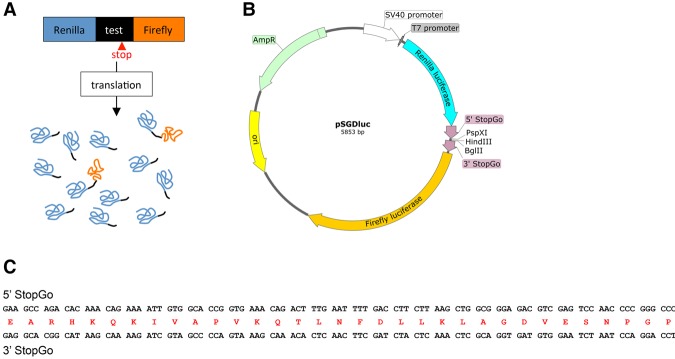
(*A*) Illustration of the traditional dual luciferase assay when used for recoding studies. Since recoding is a nonstandard translation event that is in competition with standard translation, the Renilla–Firefly luciferase fusion is often a minor product compared to the product of standard translation. Therefore, when testing recoding signals by dual luciferase assay in a single reaction, almost all Firefly luciferase activity is derived from the recoded product (Renilla–Firefly fusion), whereas Renilla luciferase activity is a combination of activities derived from both the recoded product and the product of standard translation (termination product). It is desirable to normalize Firefly luciferase activity to the activity of Renilla luciferase to minimize experimental variability caused by differences in cell viability, transfection efficiency, pipetting volumes, and cell lysis efficiency. However, this normalization step can result in discrepancies unless the Renilla luciferase activities of the termination and recoded products are similar. (*B*) Plasmid map of pSGDluc showing the 5′ and 3′ StopGo sequences flanking the polylinker sequences containing unique restriction sites for PspXI (compatible with XhoI), HindIII, and BglII (compatible with BamHI). (*C*) Nucleotide and amino acid sequences of the 5′ and 3′ FMDV StopGo sequences inserted into pSGDluc.

In the earlier versions of the dual luciferase vectors, test cassettes are inserted into a polylinker fused in-frame to the 3′ end of the sense codons for Renilla luciferase and at its 3′ end to Firefly luciferase. Ribosomes that perform the recoding event synthesize a Renilla–Firefly fusion protein, whereas those that do not, yield Renilla alone (Fig. 1A). Calculating recoding efficiency involves relative luciferase activities, taking into account activities from control constructs where Renilla–Firefly fusion does not involve the nonstandard decoding event (in-frame controls). Normalization of reporter activity values derived from decoding both before and after the recoding site obviates the effect of experimental variability caused by differences in cell viability, transfection efficiency, pipetting volumes, and cell lysis efficiency. However, this normalization can be misleading when cassette sequence product influences reporter activity or stability.

The new vector, pSGDluc, has one StopGo sequence at the 3′ end of the sequence encoding Renilla luciferase and another at the 5′ end of the sequence encoding Firefly luciferase (Fig. 1B,C).

### Comparison of pSGDluc with pDluc

**FIGURE 2.**
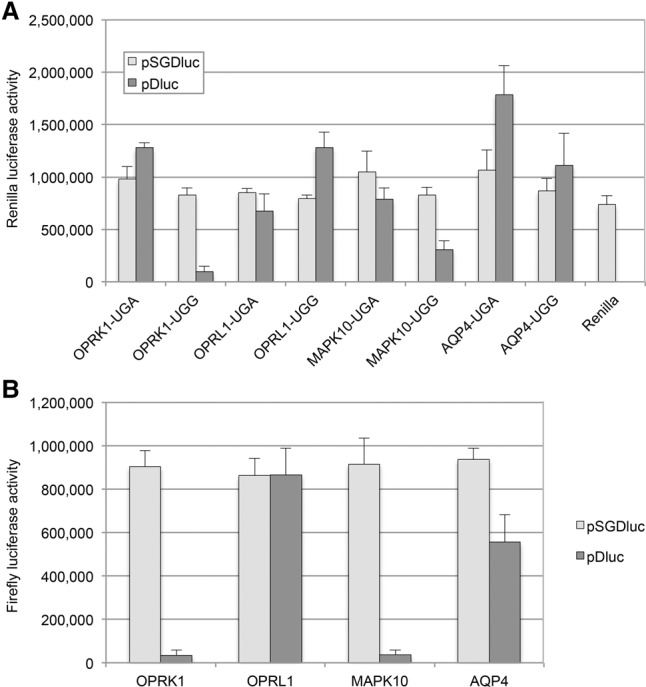
(*A*) Absolute Renilla activities determined by luciferase assay after transfection of HEK293T cells with either pSGDluc (light gray) or pDluc (dark gray) harboring readthrough signals (UGA) from human *OPRK1*, *OPRL1*, *MAPK10*, and *AQP4*, as indicated. In-frame control constructs are indicated as UGG. (*B*) Absolute Firefly activities determined by luciferase assay after transfection of HEK293T cells with either pSGDluc (light gray) or pDluc (dark gray) harboring in-frame control (UGA to UGG) sequences for human *OPRK1*, *OPRL1*, *MAPK10*, and *AQP4*, as indicated.

With pSGDluc the Renilla and Firefly activities should remain unaffected regardless of the size or amino acid composition of the proteins encoded by the recoding cassette. Therefore, Firefly activities should faithfully reflect Firefly abundance which in turn can be reliably normalized to Renilla activity to provide a more accurate estimate of recoding efficiency. To assess this prediction, we tested in pSGDluc, several known human stop codon readthrough signals that had previously been identified by comparative genomics analysis (Jungreis et al. 2011; Lindblad-Toh et al. 2011; Loughran et al. 2014). These were compared against the traditional vector system, pDluc (Fixsen and Howard 2010) (a derivative of p2luc [Grentzmann et al. 1998]). Absolute Renilla activities expressed from pSGDluc fluctuate much less than those expressed from pDluc (Fig. 2A). A similar disparity between pSGDluc and pDluc is also evident for absolute Firefly activities expressed from the in-frame controls where UGA stop codons are mutated to UGG (Fig. 2B). The StopGo reaction necessarily leaves 32 residual FMDV amino acids at the Renilla C terminus. This could potentially affect Renilla activity. However, StopGo-tagged Renilla derived from pSGDluc is at least as active as Renilla without the residual C-terminal tag (Fig. 2A).

**FIGURE 3.**
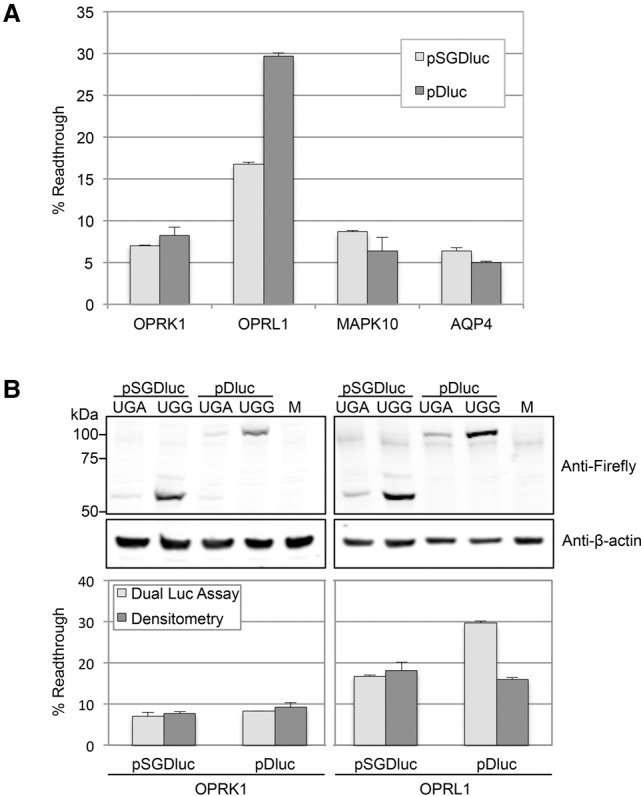
(*A*) Readthrough efficiencies determined by dual luciferase assay after transfection of HEK293T cells with either pSGDluc (light gray) or pDluc (dark gray) harboring readthrough signals from human *OPRK1*, *OPRL1*, *MAPK10*, and *AQP4*, as indicated. (*B*) Readthrough efficiencies (*lower* panel) determined by both dual luciferase assay (light gray) and densitometry (dark gray) of immunoblots (*upper* panel) after transfection of HEK293T cells with either pSGDluc or pDluc harboring readthrough signals from human *OPRK1* and *OPRL1*, as indicated. M, lysates from mock transfections.

Normalizing Firefly activities against Renilla activities and calculating readthrough efficiencies indicates readthrough levels of 17% when the readthrough cassette for the human *OPRL1* mRNA is tested in pSGDluc, whereas this level is 30% when tested in pDluc here and previously (Fig. 3A; Loughran et al. 2014). To determine whether the 17% *OPRL1* readthrough efficiency derived from pSGDluc is an accurate reflection of reporter abundance, we estimated Firefly protein levels by immunoblotting and densitometry. This indicated that the *OPRL1* readthrough efficiency determined by pSGluc (17%) is more accurate (Fig. 3B).

**FIGURE 4.**
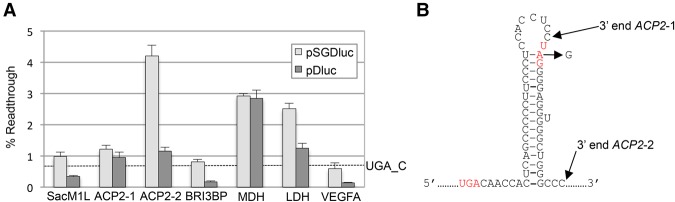
(*A*) Readthrough efficiencies determined by dual luciferase assay after transfection of HEK293T cells with either pSGDluc (light gray) or pDluc (dark gray) harboring readthrough signals from human *SACM1L, ACP2*-1*, ACP2*-2*, BRI3BP, MDH, LDH*, and *VEGFA*, as indicated. Readthrough efficiency of UGA_C cloned into pSGDluc representing background levels of readthrough on UGA_C is indicated by a dashed line. (*B*) Predicted RNA stem–loop structure immediately 3′ of the *ACP2* CDS indicating the 3′ boundaries of readthrough signals for *ACP2*-1 and *ACP2*-2. The second stop codon was mutated to UGG in construct *ACP2*-2. Stop codons are in red font.

Next we looked at all known human readthrough mRNAs for which readthrough efficiencies have been previously determined (Eswarappa et al. 2014; Loughran et al. 2014; Schueren et al. 2014; Stiebler et al. 2014). These include three mRNAs (*SACM1L*, *BRI3BP*, and *ACP2*) for which we previously could not detect levels of readthrough above 1% using pDluc (Loughran et al. 2014). For most, we see similar readthrough efficiencies between both pSGDluc and pDluc, which are in general agreement with published data (Fig. 4A). Interestingly, although for *ACP2*-1 we see a similarly low readthrough level (∼1%) from both pSGDluc and pDluc, extending the *ACP2* sequence 3′ (construct *ACP2*-2) to include a putative stem–loop structure (Fig. 4B) increases *ACP2* readthrough levels to ∼4% *only* when tested in pSGDluc. This potential readthrough stimulator was completely masked using pDluc even though identical fold increases in absolute Firefly activities between *ACP2*-1 and *ACP*2-2 were observed with both plasmids—not shown.

In contrast to the findings of a prior study (Eswarappa et al. 2014), we failed to detect readthrough levels above background (0.66%) when we tested sequences surrounding the human *VEGFA* stop codon in either plasmid (Fig. 4A). A readthrough efficiency of 9% was previously reported to generate an anti-angiogenic form of VEGF-Ax (Eswarappa et al. 2014), although this function for VEGF-Ax has recently been contested (Xin et al. 2016). The *VEGFA* sequences tested here included 10 nt 5′ and 63 nt 3′ of the *VEGFA* stop codon encompassing the putative heterogeneous nuclear ribonucleoprotein A2/B1 binding site reported to be essential for promoting *VEGFA* readthrough. One possible explanation for the discrepancy between these two studies is the different cell types used here (human HEK293T cells) and in the original study (bovine EC cells—although similar results are reported from rabbit reticulocyte lysates).

**FIGURE 5.**
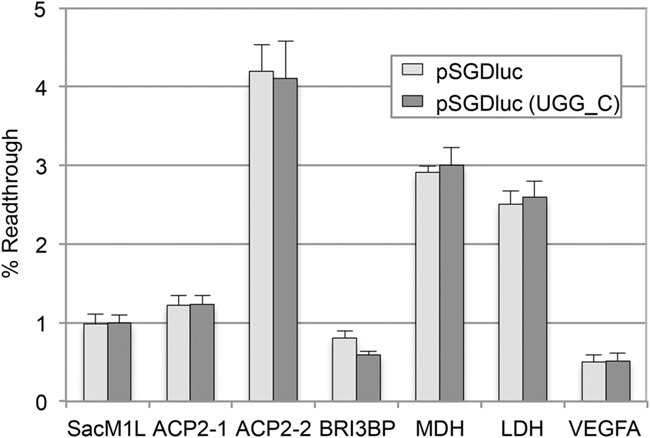
Readthrough efficiencies determined by dual luciferase assay after transfection of HEK293T cells with pSGDluc harboring readthrough signals from human *SACM1L, ACP2*-1*, ACP2*-2*, BRI3BP, MDH, LDH*, and *VEGFA*, as indicated. Normalized (Firefly/Renilla) luciferase activites were calculated for each as a percentage of their individual normalized UGG in-frame controls (light gray) or else as a percentage of a UGG_C in-frame control (dark gray).

Apart from the avoidance of potential artifactual results, we believe that pSGDluc offers several additional technical advantages over non-StopGo dual reporter plasmids. First, the number of required constructs can be reduced by half, as there is no need to generate in-frame controls for each test construct. Calculating readthrough efficiencies based on each individual in-frame control gives almost identical readthrough levels to those determined using the same in-frame control (UGG_C, Fig. 5). Furthermore, checking by immunoblotting to ensure that reporter activities and reporter abundances are in agreement, is in most cases unnecessary.

### Variants for specialized usage and caveats

Replacement of Renilla or Firefly luciferase reporters with any of the commercially available “next-generation” luciferase reporters may offer some advantages over pSGDluc in particular situations. For example, substituting Firefly with a destabilized reporter (e.g., luc2CP from Promega) could be particularly useful for the analysis of recoding events where temporal expression is being investigated. Several secreted luciferases (Gaussia and Cypridina) are available that allow measurement without cell disruption. However, it has been reported that certain nascent peptides transiting the ER translocon can inhibit the StopGo reaction (de Felipe et al. 2010).

Some caution should also be exercised if intending to use the pSGDluc system for high-throughput screening since the StopGo reaction could be inadvertently targeted, which must be controlled for. Finally, although StopGo does not function in bacteria (Donnelly et al. 1997), it does function in all eukaryotic systems tested so far including in yeast (Doronina et al. 2008; Sharma et al. 2012), insects (Diao and White 2012), and plants (Sun et al. 2012), indicating that the pSGDluc system can be easily adapted for other organisms.

## MATERIALS AND METHODS

### Plasmids

The sequence of pSGDluc has been deposited in Addgene (ID 87323). pSGDluc was generated by cloning a synthetic (Integrated DNA Technologies: g blocks) polylinker encoding tandem FMDV StopGo signals (EARHKQKIVAPV-KQTLNFDLLKLAGDVESNPGP) into plasmid pDluc, which has been previously described (Fixsen and Howard 2010). *OPRL1*, *OPRK1*, *MAPK10*, *AQP4*, *ACP2*-1, *ACP2*-2, *BRI3BP*, *SACM1L*, and *MDH* had previously been cloned into pDluc (Loughran et al. 2014). Here they were digested from pDluc with XhoI and BglII and subcloned into PspXI/BglII digested pSGDluc. *LDH* UGA and UGG constructs were generated by ligation of annealed oligonucleotide pairs 1960/1961 (UGA) and 1962/1963 (UGG) into PspXI/BglII digested pSGDluc and XhoI/BglII digested pDluc (see Table 1 for primer sequences). Renilla-only plasmid was generated by ligation of annealed oligonucleotide pairs 1964/1965 into BglII/XbaI digested pDluc. UGA_C and UGG_C constructs were generated by ligation of annealed oligonucleotide pairs 1966/1967 (UGA) and 1968/1969 (UGG) into PspXI/BglII digested pSGDluc. Sequences flanking the *VEGFA* readthrough stop codon (10 nt 5′ and 63 nt 3′) were generated by PCR on human genomic DNA using primers 1970 and 1972 for wild-type *VEGFA* and 1971 and 1972 for *VEGFA* UGG in-frame control. All *VEGFA* amplicons were digested with XhoI and BamHI and ligated into PspXI/BglII digested pSGDluc and XhoI/BglII digested pDluc. All clones were verified by sequencing.

**TABLE 1.**
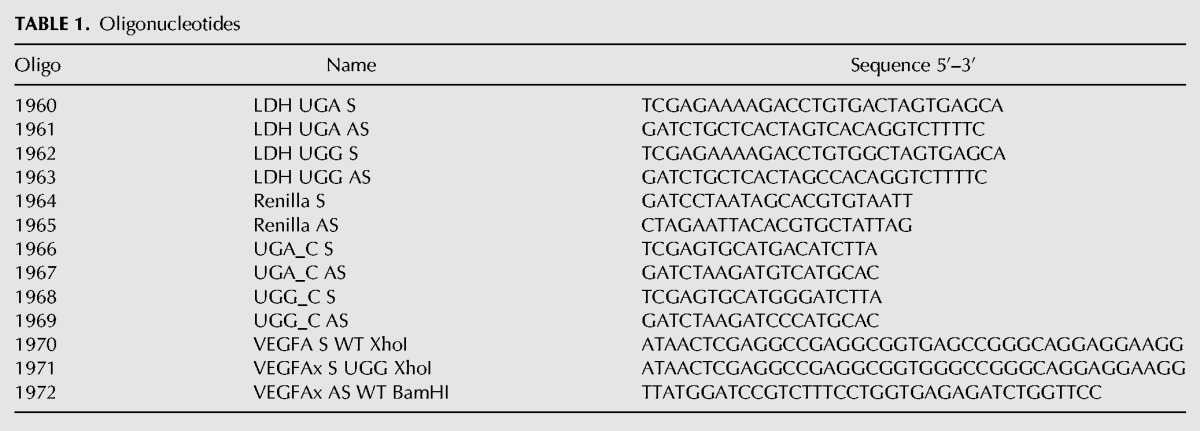
Oligonucleotides

### Cell culture and transfections

HEK293T cells (ATCC) were maintained in DMEM supplemented with 10% FBS, 1 mM l-glutamine, and antibiotics. HEK293T cells were transfected in quadruplicate with Lipofectamine 2000 reagent (Invitrogen), using the 1-d protocol in which suspended cells are added directly to the DNA complexes in half-area 96-well plates. For each transfection the following were added to each well: 25 ng of each plasmid plus 0.2 µL Lipofectamine 2000 in 25 µL Opti-Mem (Gibco). The transfecting DNA complexes in each well were incubated with 3 × 10^4^ cells suspended in 50 µL DMEM plus 10% FBS. Transfected cells were incubated at 37°C in 5% CO_2_ for 24 h.

### Dual luciferase assay

Firefly and Renilla luciferase activities were determined using the Dual Luciferase Stop & Glo Reporter Assay System (Promega). Relative light units were measured on a Veritas Microplate Luminometer with two injectors (Turner Biosystems). Transfected cells were washed once with 1× PBS and then lysed in 12.6 µL of 1× passive lysis buffer (PLB), and light emission was measured following injection of 25 µL of either Renilla or Firefly luciferase substrate. Readthrough efficiencies (percentage of readthrough) were calculated as the ratio of Firefly activity/Renilla activity for the test (UGA) sequence as a percentage of the ratio of Firefly activity/Renilla activity for the corresponding in-frame control sequence. Mean and standard deviations were calculated based on at least 12 independent transfections.

### Immunoblotting

HEK293T cells were transfected in six-well plates using Lipofectamine 2000 reagent, again using the 1-d protocol described above, with 1 µg of each indicated plasmid. The transfecting DNA complexes in each well were incubated with 1 × 10^6^ cells suspended in 3000 µL DMEM plus 10% FBS and incubated overnight at 37°C in 5% CO_2_. Transfected cells were lysed in 100 µL 1× PLB and 10 µL each lysate assayed by dual luciferase assay. Proteins were resolved by SDS–PAGE and transferred to nitrocellulose membranes (Protran), which were incubated at 4°C overnight with goat anti-Firefly (Promega) and mouse anti-β-actin (Sigma). Immunoreactive bands were detected on membranes after incubation with appropriate fluorescently labeled secondary antibody using a LI-COR Odyssey Infrared Imaging Scanner. ImageStudio software was used for densitometry. Firefly intensities were calculated relative to β-actin intensities and readthrough efficiencies determined as a percent of the corresponding normalized in-frame controls. Mean and standard deviations of relative protein intensities were determined from three biological replicates.
